# VX-445 (elexacaftor) inhibits chloride secretion across human bronchial epithelial cells by directly blocking KCa3.1 channels

**DOI:** 10.1093/pnasnexus/pgaf211

**Published:** 2025-07-04

**Authors:** Aaron Kolski-Andreaco, Corina M Balut, Matthew D Green, John Sembrat, Robert J Bridges, Ashvani K Singh, Chris Tse, Michael B Butterworth, Daniel C Devor

**Affiliations:** Department of Cell Biology, University of Pittsburgh, 3500 Terrace St., Pittsburgh, PA 15261, USA; AbbVie, Inc., ZR13, Bldg. AP9A, 1 North Waukegan Road, North Chicago, IL 60064, USA; AbbVie, Inc., ZR13, Bldg. AP9A, 1 North Waukegan Road, North Chicago, IL 60064, USA; Division of Pulmonary, Allergy, Critical Care and Sleep Medicine (PACCSM), University of Pittsburgh, 3500 Terrace St., Pittsburgh, PA 15213, USA; Department of Physiology and Biophysics, Chicago Medical School, 3333 Green Bay Rd., North Chicago, IL 60064, USA; AbbVie, Inc., ZR13, Bldg. AP9A, 1 North Waukegan Road, North Chicago, IL 60064, USA; AbbVie, Inc., ZR13, Bldg. AP9A, 1 North Waukegan Road, North Chicago, IL 60064, USA; Department of Cell Biology, University of Pittsburgh, 3500 Terrace St., Pittsburgh, PA 15261, USA; Department of Cell Biology, University of Pittsburgh, 3500 Terrace St., Pittsburgh, PA 15261, USA

**Keywords:** cystic fibrosis, ion channels, adverse events, Major Classification: Biological Sciences, Minor Classification: Pharmacology

## Abstract

Cystic fibrosis (CF) is a genetic disorder resulting from mutations to the CF transmembrane regulator (CFTR) anion channel. CFTR correctors partially restore the folding and trafficking of mutant CFTR. We recently demonstrated that the correctors VX-445 and VX-121 directly potentiate large-conductance Ca^2+^-activated (BK_Ca_) channels. We postulated that this could enhance the therapeutic potential of these drugs in the lung by increasing the driving force for transepithelial Cl^−^ secretion. Herein, we evaluated the effect of acute addition of VX-445 on forskolin- and 5,6-dichloro-1-ethyl-1,3-dihydro-2H-benzimidazol-2-one-mediated Cl^−^ secretion across primary human bronchial epithelial cells (HBEs) from wild type (WT) and F508del donors. Surprisingly, VX-445 (10 µM) induced a significant inhibition of forskolin-stimulated Cl^−^ secretion in WT and F508del donor HBEs with corrected CFTR. We hypothesized that this was due to inhibition of the basolateral membrane Ca^2+^-activated K^+^ channel, KCa3.1 that maintains the driving force for Cl^−^ secretion. Thus, we utilized patch-clamp techniques to evaluate VX-445 effects on isolated KCa3.1 currents. We demonstrate that VX-445 directly inhibits KCa3.1, as do similar molecules VX-659 and VX-121; however, only VX-659 inhibited KCa2.3 and KCa2.2 with a similar affinity to KCa3.1. To summarize, acute addition of CFTR correctors to HBEs reduces transepithelial Cl^−^ secretion due to inhibition of KCa3.1.

Significance Statement Here, we show, for the first time, that class 2 (C2) cystic fibrosis (CF) transmembrane regulator (CFTR) correctors paradoxically inhibit forskolin- and 5,6-dichloro-1-ethyl-1,3-dihydro-2H-benzimidazol-2-one-stimulated Cl^−^ secretion across wild type (WT) and F508del CFTR-expressing human bronchial epithelial cells. We demonstrate that this effect is due to the direct inhibition of basolateral KCa3.1 by the standard-of-care C2 corrector VX-445. This work provides potential insight into adverse events experienced by patients with CF on elexacaftor, tezacaftor, and ivacaftor therapy.

## Introduction

Cystic fibrosis (CF) is a devastating genetic disorder affecting ∼100,000 people worldwide (1). The disease is characterized by defects in Cl^−^, ${\mathrm{HCO}}_{3}^{-}$, and Na^+^ transport, which affects epithelia—most recognizably, the lungs (2). In the respiratory system, dysfunctional Cl^−^ secretion leads to a reduction in the airway surface liquid (ASL) height, resulting in reduced mucociliary clearance, and a dramatic increase in the risk of infection (3–6). Underlying the pathophysiology of CF are mutations in the CF transmembrane conductance regulator (CFTR) protein—an apical membrane Cl^−^ channel in epithelia. These mutations are separated into six unique categories, based on how they result in a loss of CFTR expression/function (7). The two most common categories of mutations include those that affect either the gating or trafficking of CFTR. F508del, the result of a deleted phenylalanine at amino acid position 508, which is expressed in ∼85% of patients with CF, results in a misfolded protein that fails to traffic correctly to the plasma membrane. Therapeutics that target folding/trafficking mutations are known as “correctors.” On the contrary, the missense mutations, of which G551D is the most common, occurring in ∼5% of patients with CF, results in normal trafficking of a mutated CFTR channel with abnormal channel gating. Appropriately, drugs that target gating mutations in CF are referred to as “potentiators.”

Following the discovery that CF epithelial function could be rescued by a small molecule in vitro (8), VX-770 or ivacaftor emerged as the first clinically approved potentiator therapy for CF—a drug that increases the open probability (*P*_o_) of CFTR. However, VX-770 targets gating mutations only, such that it had little efficacy in F508del patients. More recently, both class 1 (C1; VX-661 or tezacaftor) and class 2 (C2; VX-445 or elexacaftor) correctors were developed to partially correct the folding/trafficking of F508del CFTR. The combination of elexacaftor, tezacaftor, and ivacaftor (ETI) was FDA-approved in 2019 as Trikafta and has proven incredibly efficacious in improving lung function of patient with CF and hence their quality of life (9).

We previously demonstrated that potentiators of basolateral KCa3.1 (KCNN4) including 5,6-dichloro-1-ethyl-1,3-dihydro-2H-benzimidazol-2-one (DCEBIO) stimulate Cl^−^ secretion across both wild type (WT) and F508del CFTR-expressing HBEs (10–13). Based on this, we postulated that modulation of K^+^ channels could be therapeutically beneficial in CF. Recently, we demonstrated that the C2 CFTR correctors VX-445 and VX-121 act as direct potentiators of apical BK_Ca_ (K_Ca_1.1, KCNMA1) channels in primary HBEs and in HEK cells heterologously expressing the channel (14). In contrast, other components of ETI, tezacaftor and ivacaftor, had no effect on BK_Ca_ (14). Others have similarly speculated that potentiation of BK_Ca_ would increase the driving force for Cl^−^ secretion across human airways, resulting in increased Cl^−^ secretion and hence an increased ASL volume (15–19). Herein, we directly determined whether acute addition of VX-445 potentiates the forskolin- and DCEBIO-stimulated Cl^−^ secretion across HBEs expressing WT or F508del CFTR. Paradoxically, VX-445 decreased transepithelial Cl^−^ current, whereas the C1 CFTR corrector, VX-661, had no effect.

As KCa3.1 and BK_Ca_ are both members of the broader Ca^2+^-activated K^+^ channel family, we determined whether KCa3.1 was also modulated by CFTR correctors. Using whole-cell and excised patch-clamp techniques, we demonstrate that VX-445 directly inhibits KCa3.1 channels in HBEs as well as when expressed in HEK cells. The additional C2 correctors, VX-659 and VX-121, also inhibit KCa3.1, whereas the C1 correctors, VX-661 and VX-809, had little to no effect.

In total, we demonstrate a direct inhibitory effect of VX-445 on KCa3.1 at low µM concentrations, resulting in a diminished Cl^−^ secretory response. As the concentrations utilized are known to be achieved in both plasma (20–22) and nasal turbinate cells (23), our data may explain the diminished efficacy of these compounds in a subset of patients (24). Further, as we previously demonstrated that VX-445 and VX-121 potentiate BK_Ca_ channels, this suggests a more general effect on the Ca^2+^-activated K^+^ channel gene family. As KCa3.1 and KCa2.x channels are widely expressed, our results may help to explain some of the adverse events (AE) reported (25–31).

## Results

**Fig. 1.**
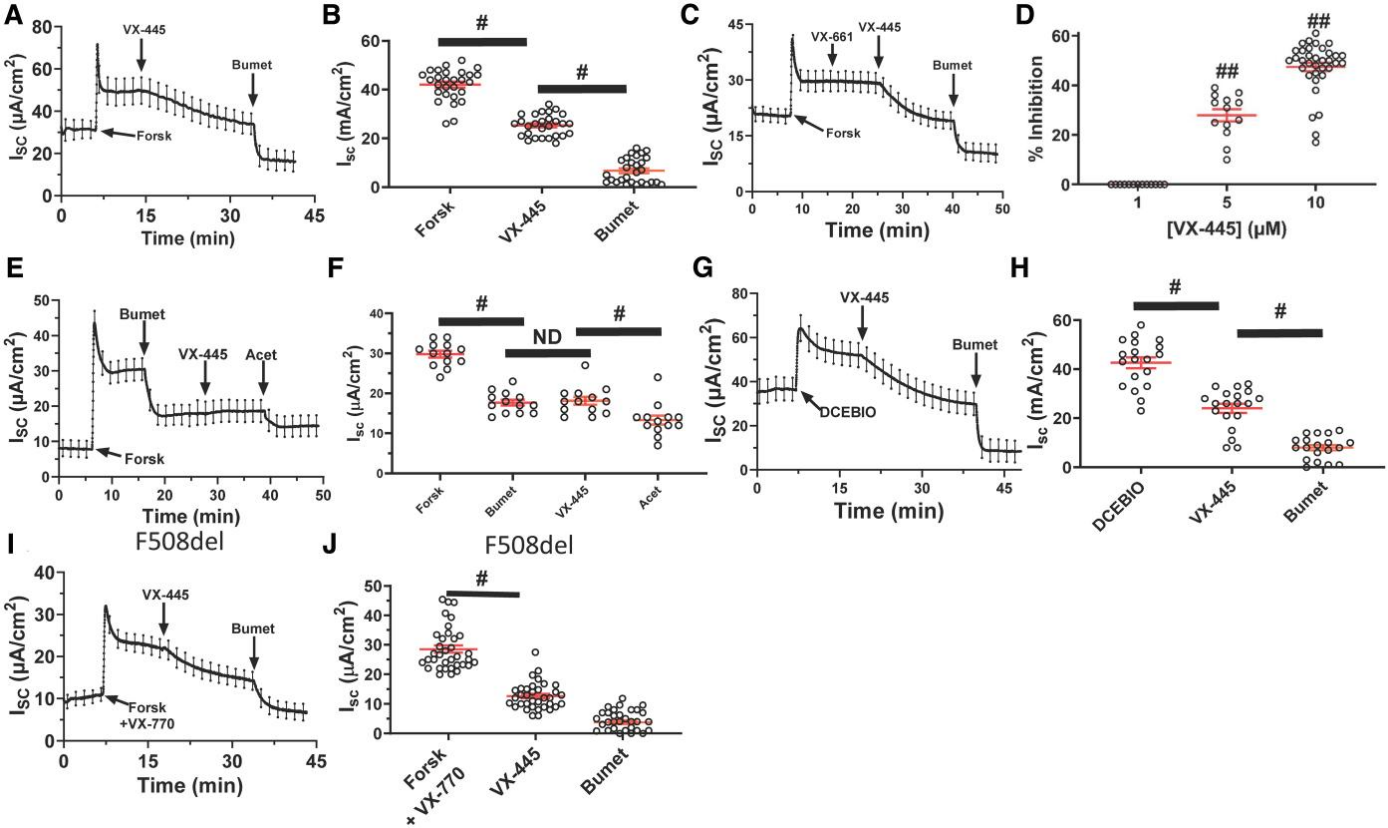
Effect of VX-445 on forskolin- and DCEBIO-induced *I*_eq_ in WT CFTR and F508del CFTR HBEs. A) In WT CFTR-expressing HBEs, forskolin stimulated a sustained *I*_sc_ that was partially inhibited by VX-445 (10 µM). B) Average *I*_sc_ in the presence of forskolin, VX-445, and bumetanide (*n* = 35). C) VX-661 (10 µM) had no effect on forskolin-stimulated current, whereas the subsequent addition of VX-445 (10 µM) inhibited *I*_sc_. D) Concentration dependence of VX-445 on *I*_sc_ expressed as percent inhibition from control. 1 µM VX-445 failed to inhibit the forskolin-stimulated current, whereas 5 µM (*P* < 0.001) and 10 µM (*P* < 0.001) demonstrated increased current inhibition. E) VX-445 (10 µM) has no effect when applied after bumetanide. Acetazolamide (1 µM) blocked bicarbonate secretion. F) Summary data showing that VX-445 does not inhibit *I*_sc_ following bumetanide, whereas acetazolamide induces a significant inhibition. G) DCEBIO (100 µM) stimulated a sustained *I*_sc_ that was partially inhibited by VX-445 (10 µM). H) Average *I*_sc_ in the presence of DCEBIO, VX-445, and bumetanide (*n* = 19). I) In corrected F508del CFTR-expressing HBEs, forskolin plus VX-770 stimulated a sustained *I*_sc_ that was partially inhibited by VX-445 (10 µM). J) Average *I*_sc_ in the presence of forskolin/VX-770, VX-445, and bumetanide. Studies were carried out on 35 filters from seven separate donors. #, *P* < 0.01; ANOVA or paired t tests. Not different (ND).

We previously demonstrated that VX-445 directly potentiates apical membrane BK_Ca_ in both WT and F508del CFTR HBEs (14). It has been proposed that, similar to potentiation of basolateral KCa3.1, potentiation of apical BK_Ca_ would further stimulate Cl^−^ secretion across HBEs by increasing the electrochemical driving force for Cl^−^ exit across the apical membrane (18, 19). To assess this possibility, we determined whether acute addition of VX-445 affects forskolin-mediated Cl^−^ secretion across primary WT CFTR-expressing HBEs, using *I*_sc_ measurements. In all of our studies, we initially inhibited Na^+^ absorption with amiloride (10 µM), although this is not shown in order to focus on the Cl^−^ secretory response. As shown in Fig. 1A, forskolin (10 µM) stimulated a sustained Cl^−^ secretory current, as expected. Surprisingly, the addition of VX-445 (10 µM) resulted in a decrease in *I*_sc_. Note, as VX-445 is a lipophilic compound, it was added to both apical and basolateral membranes. The addition of bumetanide (20 µM) further reduced *I*_sc_, as expected. In aggregate, 35 filters from five separate donors demonstrate that VX-445 decreases the forskolin-induced Cl^−^ current an average of 47 ± 2% (Fig. 1B, *P* < 0.01). In contrast to these results, VX-661 had no effect on forskolin-mediated Cl^−^ secretion, while the subsequent addition of VX-445 decreased *I*_sc_, as above (Fig. 1C). In a total of nine filters from three separate donors, VX-445 inhibited an average of 47 ± 2% of the current, subsequent to VX-661 (*P* < 0.01). The concentration dependence of the VX-445 inhibition is shown in Fig. 1D. While 1 µM VX-445 had no effect, both 5 µM and 10 µM VX-445 significantly decreased the forskolin-mediated Cl^−^ secretory response (*P* < 0.001). The current observed following forskolin stimulation consists of both bumetanide-sensitive Cl^−^ secretion and bumetanide-insensitive ${\mathrm{HCO}}_{3}^{-}$ secretion (32–34). Thus, we determined whether VX-445 would inhibit the remaining current subsequent to bumetanide. As shown in Fig. 1E, VX-445 (10 µM) had no effect on the bumetanide-insensitive current. However, the subsequent addition of the carbonic anhydrase inhibitor, acetazolamide (1 µM), partially inhibited the remaining current, confirming that this is due to ${\mathrm{HCO}}_{3}^{-}$ secretion. The average response from two separate donors and 13 filters is shown in Fig. 1F. Given the surprising inhibition of Cl^−^ secretion observed, we chose to stimulate Cl^−^ secretion via an alternate mechanism and assess the effect of VX-445. We previously demonstrated that DCEBIO stimulates Cl^−^ secretion, via activation of KCa3.1, in both WT and CF HBEs (12, 13). Thus, we determined the effect of VX-445 on the DCEBIO-induced Cl^−^ secretory response. As illustrated in Fig. 1G and H, VX-445 (10 µM) decreased the DCEBIO-induced Cl^−^ current an average of 54 ± 2% (*n* = 19; *P* < 0.01). We next determined whether VX-445 would similarly decrease the forskolin-stimulated transepithelial Cl^−^ currents in corrected F508del HBEs. F508del CFTR was corrected by overnight incubation with VX-445 (1 µM) plus VX-661 (3 µM). As shown above, these concentrations of VX-445 and VX-661 had no acute effect on Cl^−^ secretion. The subsequent addition of forskolin plus VX-770 (1 µM) evoked a sustained Cl^−^ secretory current, as described (Fig. 1I) (12). The subsequent acute addition of 10 µM VX-445 induced a decrease in *I*_sc_, similar to what was observed in WT CFTR HBEs. The addition of bumetanide further inhibited *I*_sc_, as expected. In a total of 35 filters from five separate donors, this decrease averaged 64 ± 2% (*P* < 0.01; Fig. 1J).

**Fig. 2.**
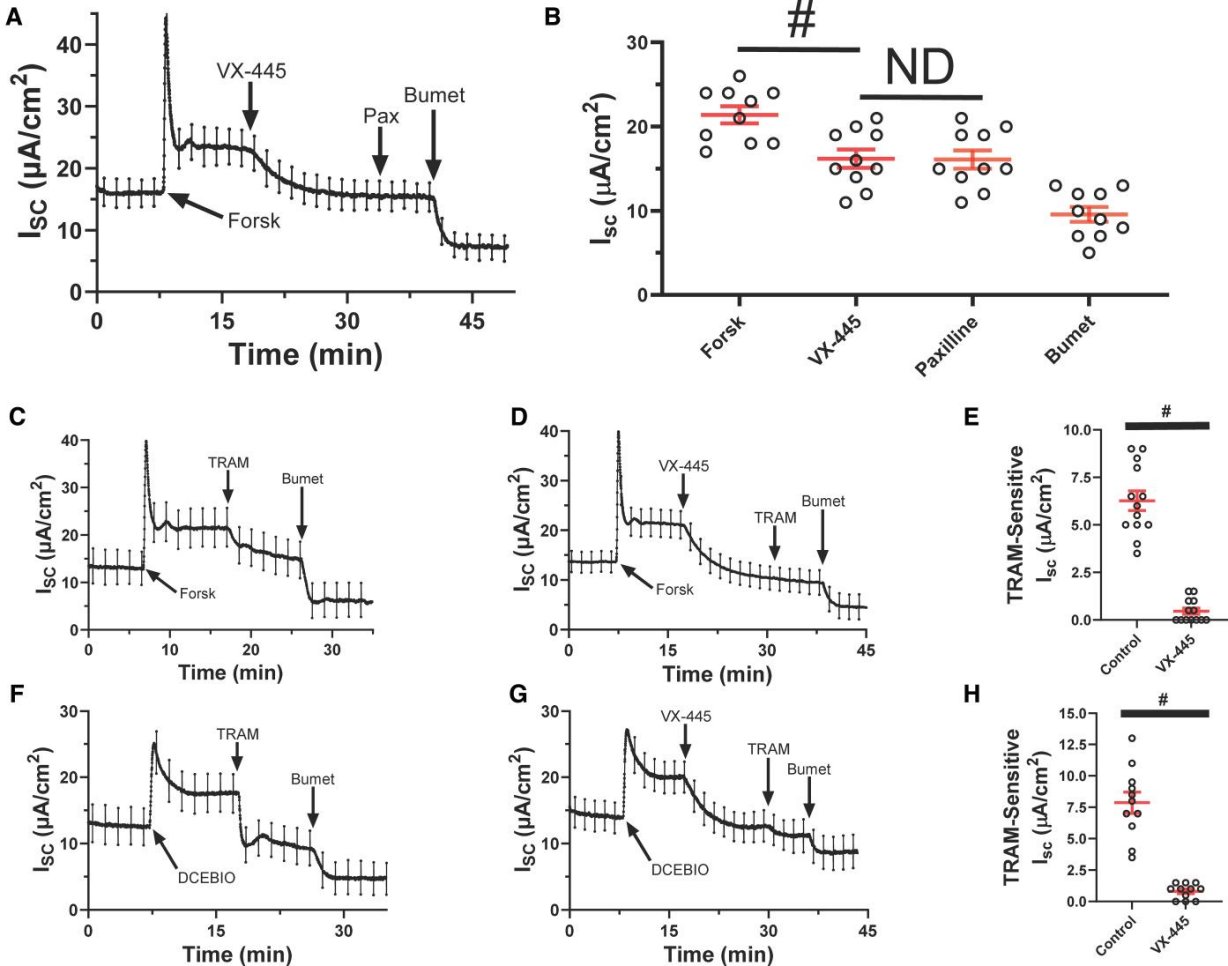
Effect of K^+^ channel inhibitors following VX-445 inhibition of *I*_sc_ in WT CFTR HBEs. Forskolin stimulated a sustained *I*_sc_ that was partially inhibited by VX-445 (10 µM). The subsequent addition of paxilline (Pax, 10 µM) had no effect on *I*_sc_. B) Average *I*_sc_ in the presence of forskolin, VX-445, paxilline, and bumetanide (*n* = 16) from three separate donors. #, *P* < 0.01, not different (ND), ANOVA. C) Forskolin stimulated a sustained *I*_sc_ that was partially inhibited by TRAM-34 (5 µM). D) Forskolin stimulated a sustained *I*_sc_ that was partially inhibited by VX-445 (10 µM). The subsequent addition of TRAM-34 had little effect. E) Average TRAM-sensitive *I*_sc_ for 13 filters in the presence of forskolin (control) or following VX-445. F) DCEBIO (100 µM) stimulated a sustained *I*_sc_ that was partially inhibited by TRAM-34 (5 µM). G) DCEBIO stimulated a sustained *I*_sc_ that was partially inhibited by VX-445 (10 µM). The subsequent addition of TRAM-34 had little effect. H) Average TRAM-sensitive *I*_sc_ for 11 filters in the presence of DCEBIO (control) or following VX-445. #, *P* < 0.01, unpaired t test. Data from four separate donors.

We recently demonstrated that VX-445 directly potentiates the apical BK_Ca_ channel in HBEs (14). Thus, one possibility to explain the decrease in *I*_sc_ observed is the stimulation of apical K^+^ secretion by VX-445, which would result in an inwardly directed current. In this case, the subsequent addition of a BK_Ca_ channel blocker should reverse the effect of VX-445, resulting in an apparent increase in *I*_sc_. However, as shown in Fig. 2A, subsequent to the VX-445-induced decrease in forskolin-stimulated *I*_sc_, further addition of the BK_Ca_ inhibitor, paxilline (10 µM), had no effect on *I*_sc_, whereas bumetanide inhibited the current, as expected. The average data for 16 experiments from three separate donors are shown in Fig. 2B. As above, VX-445 inhibited an average of 45 ± 2% of the forskolin-stimulated *I*_sc_. This result argues against the possibility that the decrease in *I*_sc_ observed with VX-445 is due to the stimulation of K^+^ secretion. A second possibility is, rather paradoxically, that VX-445 is inhibiting Cl^−^ secretion. As VX-445 inhibits both forskolin- and DCEBIO-induced Cl^−^ secretion, the most parsimonious explanation is that the same target is being affected in both instances. We previously demonstrated that basolateral KCa3.1 is critical for maintaining both the forskolin- and DCEBIO-mediated Cl^−^ secretory response in HBEs (12). Thus, we hypothesized that VX-445 may affect KCa3.1 gating to modulate the Cl^−^ secretory response. To begin to address this, we determined whether VX-445 alters the TRAM-34-sensitive current, as TRAM-34 is a selective KCa3.1 inhibitor. As shown in Fig. 2C, following forskolin stimulation of *I*_sc_, TRAM-34 inhibits a portion of the current, consistent with KCa3.1 playing a role in this response, as we previously demonstrated (12). In contrast, as shown in Fig. 2D, following VX-445-induced inhibition of *I*_sc_, TRAM-34 failed to inhibit additional current. In the four unique donors relative to those above, VX-445 inhibited an average of 56 ± 2% of the forskolin-stimulated current (*n* = 13; *P* < 0.01). The average change in current following TRAM-34 in the absence and presence of VX-445 for these four donors is shown in Fig. 2E. Similar studies were carried out following stimulation of Cl^−^ secretion by the KCa3.1 potentiator, DCEBIO (Fig. 2D–F). As shown in Fig. 2F, TRAM-34 inhibits the DCEBIO-stimulated *I*_sc_ response, as we previously demonstrated (10, 12, 13, 35, 36). As shown in Fig. 2G and H, the magnitude of the TRAM-34-sensitive current decreases following VX-445-induced inhibition of *I*_sc_. In these four donors, VX-445 inhibited an average of 69 ± 3% (*n* = 12; *P* < 0.01) of the DCEBIO-induced current. These results are consistent with VX-445 inhibiting KCa3.1, thereby diminishing the magnitude of TRAM-34 inhibition. As VX-445 did not inhibit the bumetanide-insensitive current, we determined whether this current was dependent upon KCa3.1. In 10 filters from two donors, TRAM-34 failed to decrease the bumetanide-insensitive current, indicating that KCa3.1 does not play a role in this current (data not shown). This further suggests that VX-445 inhibits KCa3.1 as a means of decreasing transepithelial Cl^−^ secretion across HBEs.

**Fig. 3.**
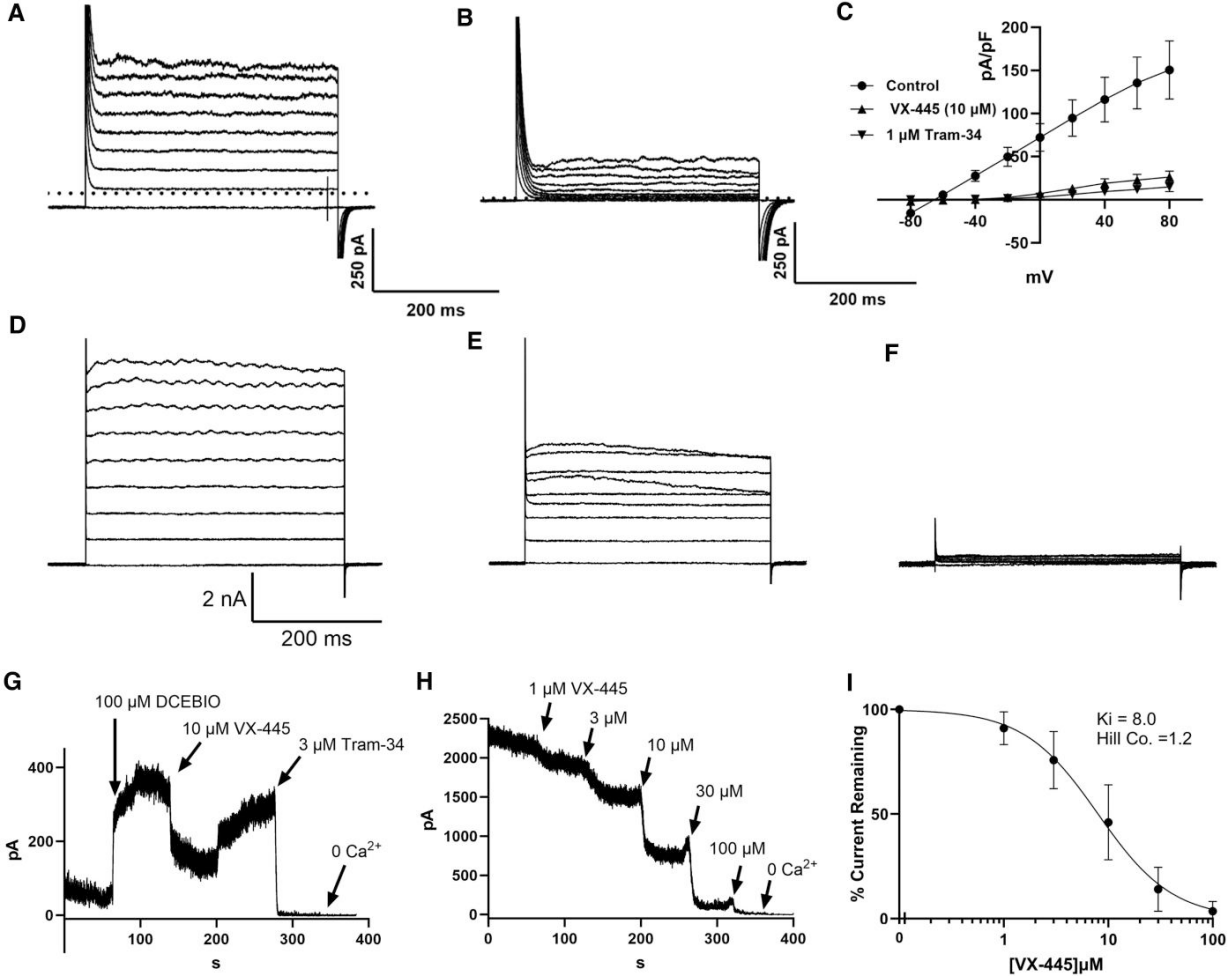
Inhibition of endogenous and heterologous KCa3.1 currents by VX-445. Representative whole-cell currents from a nonpolarized, undifferentiated HBE cell held at −80 mV and pulsed to 80 mV in 20 mV increments having a pipette free Ca^2+^ of 500 nM under control conditions (A) and exposed to 10 µM VX-445 (B). C) Average *I–V* relationship, normalized to cell capacitance, for HBEs exposed to VX-445 and subsequently TRAM-34 (*n* = 3). D) Whole-cell current recording in control conditions from a HEK cell heterologously expressing KCa3.1. The cell was then exposed to 10 µM VX-445 (E) and TRAM-34 (F). G) Following the establishment of the inside-out patch configuration in 500 nM free Ca^2+^, KCa3.1 channel activity was further increased by exposure to 100 µM DCEBIO. The subsequent addition of 10 µM VX-445 reversibly inhibited KCa3.1. The addition of 3 µM TRAM-34 and 0 Ca^2+^ inhibited the remainder of the KCa3.1 current. H) Patch was excised into 3 µM free Ca^2+^ and subsequently exposed to increasing concentrations of VX-445 before 0 Ca^2+^ was added to establish baseline. I) Average current inhibition from four experiments performed as in E). Data were fitted to the Hill equation with an apparent *K*_i_ of 8.0 µM with a Hill coefficient of 1.2.

Our *I*_sc_ results are consistent with VX-445 inhibiting KCa3.1 in HBEs. To directly assess this possibility, we initially carried out whole-cell patch-clamp studies on undifferentiated HBEs. When 500 nM free Ca^2+^ is present in the pipette solution, these cells exhibit robust outward potassium currents in response to voltage pulses from −80 to 80 mV (Fig. 3A). Upon application of 10 µM VX-445, a majority of this current was inhibited, averaging 87 ± 4% (*n* = 3, *P* < 0.005; Fig. 3B). A summary of the current–voltage (*I–V*) relationship—normalized to cell capacitance—is shown in Fig. 3C, which also includes the whole-cell *I–V* in the presence of Tram-34. These results demonstrate that VX-445 inhibits endogenous KCa3.1 in WT CFTR-expressing HBEs. To ensure isolation of KCa3.1 currents and to more fully characterize this VX-445-dependent inhibition of KCa3.1, we utilized HEK cells heterologously expressing KCa3.1 (HEK-KCa3.1). Initially, we confirmed our results from HBEs, using whole-cell recordings, as above. HEK-KCa3.1 cells exhibited large outward currents (Fig. 3D), which were inhibited by 10 µM VX-445 (Fig. 3E). In seven experiments, this inhibition averaged 58 ± 7% (*P* < 0.005). The subsequent addition of TRAM-34 (1 µM) inhibited the remainder of the current, confirming the expression of KCa3.1 (3F). It is well recognized that drugs are carried to their targets in the blood bound to albumin, which ranges from 3.4 to 5.4%. Thus, we determined whether VX-445 (10 µM) would inhibit KCa3.1 expressed in HEK cells in the presence of 3.4% albumin. In four experiments, VX-445 inhibited an average of 51 ± 17% of the whole-cell KCa3.1 current, which is not different from that observed in the absence of albumin. To confirm the direct effect of VX-445 on KCa3.1, we utilized the excised, inside-out patch-clamp technique. The patches were excised into 1 µM free Ca^2+^ to ensure baseline channel activity, as shown in Fig. 3G. The patch was then exposed to DCEBIO (100 µM) to further activate the channels (34–37)—analogous to our *I*_sc_ studies in HBEs. Importantly, VX-445 (10 µM) partially inhibited KCa3.1 channel function in a reversible manner. The subsequent addition of TRAM-34 (3 µM) inhibited the remainder of the current, consistent with KCa3.1 expression (Fig. 3G). In three experiments, VX-445 inhibited an average of 63 ± 6% of the KCa3.1 current. Based on these experiments, we determined the apparent inhibitory constant, *K*_i_ for VX-445. For these experiments, patches were excised into saturating Ca^2+^ (3 µM) to ensure maximal KCa3.1 activation. These studies were carried out in the absence of DCEBIO to eliminate the possibility that the inhibition observed was due to competition for the DCEBIO-binding site. As shown in Fig. 3H, increasing concentrations of VX-445 resulted in a near-complete inhibition of KCa3.1 channel activity. These data were fitted to the Hill equation, yielding an apparent *K*_i_ of 8.0 ± 2.0 µM with a Hill coefficient of 1.2 (Fig. 3I; *n* = 4). These data demonstrate that the current standard-of-care (SOC) C2 CFTR corrector, VX-445, directly inhibits KCa3.1.

As part of Trikafta, VX-445 is combined with a C1 CFTR corrector, VX-661, with a unique structure and binding site. We demonstrate that VX-661 has no effect on forskolin-stimulated Cl^−^ secretion across WT CFTR-expressing HBEs. In whole-cell patch-clamp recordings, we confirmed that VX-661 had no effect on KCa3.1 current (10 µM, not shown). Similarly, the older generation C1 corrector, VX-809, failed to inhibit KCa3.1 at 10 µM (not shown).

**Fig. 4.**
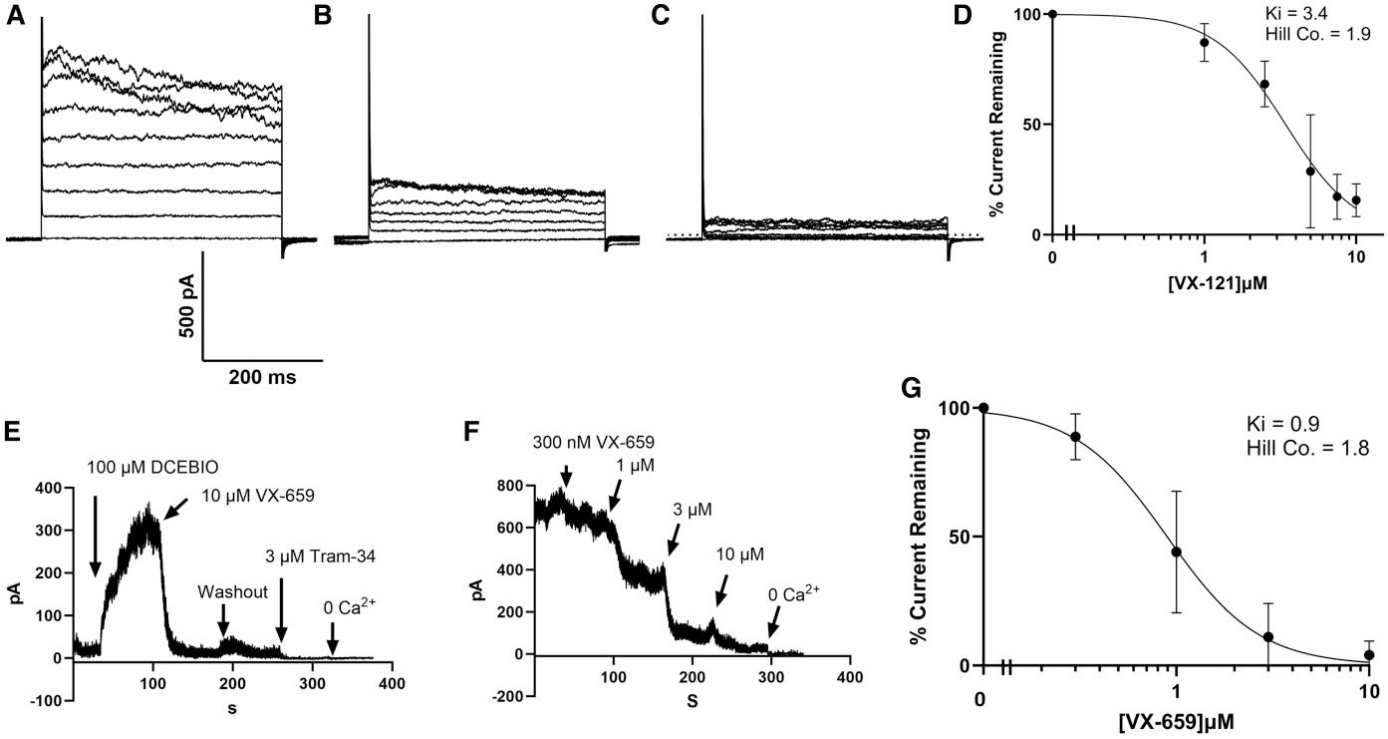
Effect of VX-121 and VX-659 on KCa3.1 heterologously expressed in HEK Cells. A) Control whole-cell recording demonstrating outward currents in response to voltage pulses from −80 to +80 mV in 20 mV increments. B) Same cell exposed to 10 µM VX-121. C) Same cell in the presence of 5 µM TRAM-34. D) Hill plot showing concentration–response relationship of VX-121 on KCa3.1 fit to the Hill equation. The apparent *K*_i_ was 3.4 µM with a Hill coefficient of 1.8. E) Following the establishment of the inside-out patch configuration in 500 nM free Ca^2+^, KCa3.1 channel activity was further increased by exposure to 100 µM DCEBIO. The subsequent addition of 10 µM VX-659 inhibited KCa3.1, and this was poorly reversible. The addition of 3 µM TRAM-34 and 0 Ca^2+^ inhibited the remainder of the KCa3.1 current. F) Patch was excised into 3 µM free Ca^2+^ and subsequently exposed to increasing concentrations of VX-659 before 0 Ca^2+^ was added to establish baseline. G) Average of four experiments performed as in F). Data were fitted to the Hill equation with an apparent *K*_i_ of 0.89 ± 0.14 µM with a Hill coefficient of 1.8.

Recently, Vertex developed the next-generation C2 corrector VX-121 (vanzacaftor). We demonstrated that VX-121 similarly potentiates BK_Ca_ in HBEs and the alpha subunit itself (14). Based on this, we determined whether VX-121 would similarly inhibit KCa3.1 expressed in HEK cells. As shown in Fig. 4A, robust whole-cell currents were observed under control condition, and these were significantly inhibited by VX-121 (10 µM, Fig. 4B). In three experiments, this inhibition averaged 85 ± 8%. The subsequent addition of TRAM-34 completely inhibited the KCa3.1 currents (4C). Concentration–response data from 17 experiments are shown in Fig. 4D and reveal an apparent *K*_i_ of 3.4 µM and a Hill coefficient of 1.9. Given that both C2 correctors, VX-445 and VX-121, inhibit KCa3.1, we extended our studies to determine whether the additional C2 corrector, VX-659 (bamocaftor), would similarly inhibit KCa3.1, as we demonstrated that this compound weakly potentiates BK_Ca_ (14). As shown in Fig. 4E, following excision of the patch into 1 µM free Ca^2+^ and stimulation with DCEBIO (100 µM), the subsequent addition of 10 µM VX-659 induced an almost complete block of KCa3.1 currents, which was difficult to reverse. The remaining current was inhibited by TRAM-34. The addition of 0 Ca^2+^ (0 added Ca^2+^ plus 1 mM EGTA) was used to establish the zero current level. In three experiments, this inhibition averaged 98 ± 1%. To determine the concentration dependence of this inhibition, we carried out studies in 3 µM Ca^2+^, in the absence of DCEBIO, as above. As shown in Fig. 4F, VX-659 inhibited KCa3.1 in a concentration-dependent manner. The relative currents remaining were fit to the Hill equation yielding an apparent *K*_i_ of 0.89 ± 0.14 µM with a Hill coefficient of 1.8 (Fig. 4G; *n* = 4). These data demonstrate that the CFTR corrector, VX-659, is a significantly higher affinity inhibitor of KCa3.1 than the structurally similar VX-445.

KCa3.1 belongs to the KCNNX gene family, of which there are three additional members, KCa2.1–KCa2.3. Indeed, KCa3.1 shares ∼40% sequence similarity with the KCa2.x channels, which accounts for the gating mechanism they share. However, KCa3.1 and KCa2.1–3 homotetramers are easily distinguished from each other pharmacologically, exhibiting unique blocker profiles. Based on this, we determined whether CFTR correctors also inhibit KCa2.x (SK) currents.

**Fig. 5.**
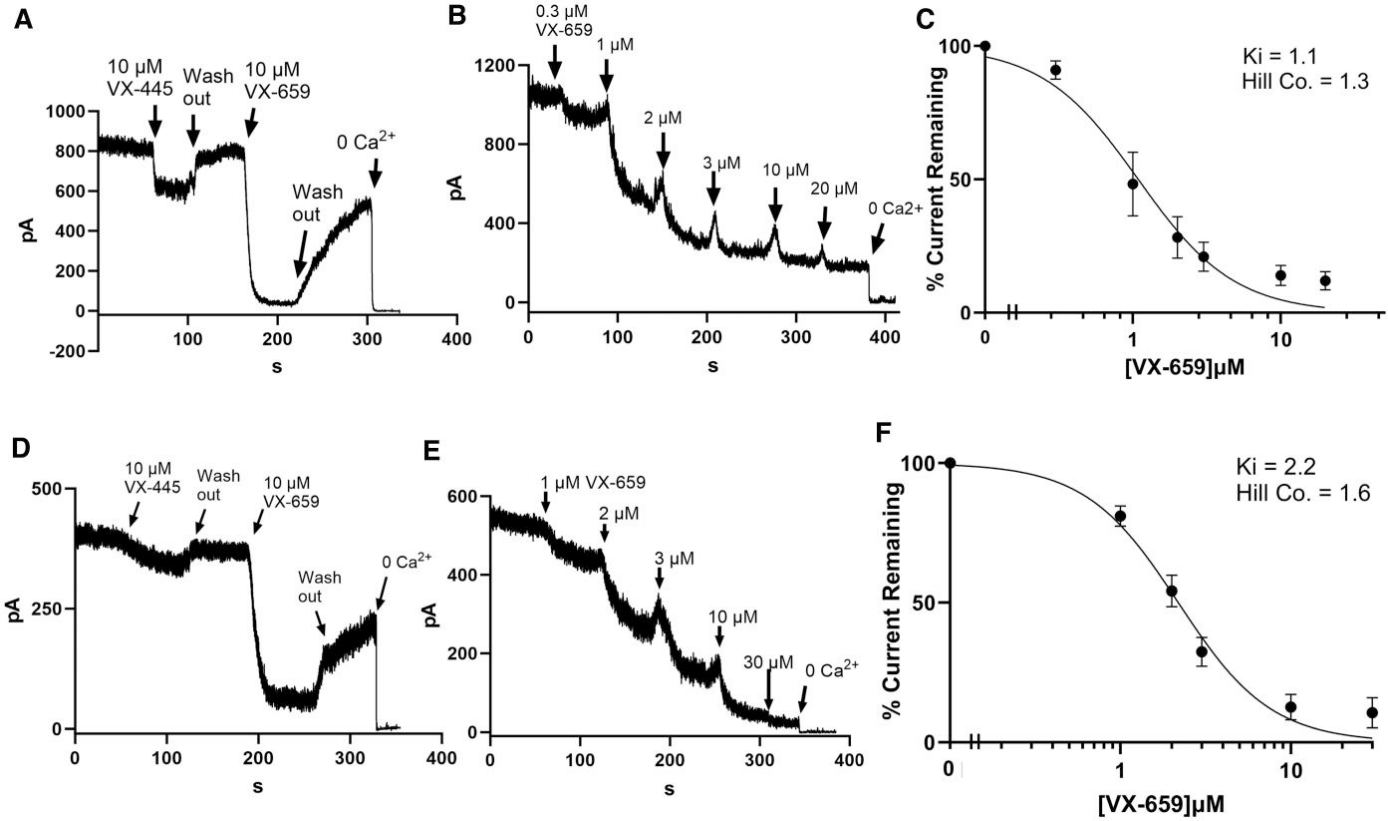
Effects of C2 correctors on HEK cells heterologously expressing KCa2.X (SK) channels. A) Following the establishment of the inside-out patch configuration in 1 µM free Ca^2+^, significant KCa2.3 channel activity was observed. Exposure to 10 µM VX-445 induced a small inhibition of KCa2.3 (15 ± 5%, *n* = 4). The subsequent addition of 10 µM VX-659 nearly completely inhibited KCa2.3 (91 ± 3%, *n* = 4), and this was partially reversible. The addition of 0 Ca^2+^ inhibited the remainder of the current. B) Patch was excised into 1 µM free Ca^2+^ and subsequently exposed to increasing concentrations of VX-659 before 0 Ca^2+^ was added to establish baseline. C) Average of five experiments performed as in B). Data were fitted to the Hill equation with an apparent *K*_i_ of 0.77 ± 0.07 µM and a Hill coefficient of 1.9. D) Following patch excision into 1 µM free Ca^2+^, significant current was observed. Exposure to 10 µM VX-445 induced a small, reversible inhibition of KCa2.2 (10 ± 4%, *n* = 4). The subsequent addition of 10 µM VX-659 significantly inhibited KCa2.2 (74 ± 4%, *n* = 4), and this was partially reversible. The addition of 0 Ca^2+^ inhibited the remainder of the current. E) Patch was excised into 1 µM free Ca^2+^ and subsequently exposed to increasing concentrations of VX-659 before 0 Ca^2+^ was added to establish baseline. F) Average of six experiments performed as in E). Data were fitted to the Hill equation with an apparent *K*_i_ of 1.95 ± 0.19 µM and a Hill coefficient of 2.1.

As above, we performed excised patch-clamp recordings on HEK cells heterologously expressing KCa2.3 or KCa2.2, in order to assess the activity of C2 CFTR corrector compounds. As shown in Fig. 5A, 10 µM VX-445 produced a small, reversible inhibition of KCa2.3 currents, which averaged 15 ± 5% (*n* = 4). In contrast, the subsequent addition of 10 µM VX-659 yielded an almost complete inhibition of KCa2.3 currents, averaging 91 ± 3% (*n* = 4). As shown in Fig. 5B, VX-659 induced a concentration-dependent inhibition of KCa2.3. These data were fitted to the Hill equation, yielding an apparent *K*_i_ of 1.1 ± 0.1 µM (Fig. 5C; *n* = 5). Finally, we determined whether KCa2.2 would be similarly inhibited. As shown in Fig. 5D, 10 µM VX-445 induced a small, reversible inhibition of KCa2.2, averaging 10 ± 4% (*n* = 4), whereas the subsequent addition of 10 µM VX-659 inhibited an average of 74 ± 4% (*n* = 4) of KCa2.2 channel current. A complete concentration–response study (Fig. 5E) yielded an apparent *K*_i_ of 1.9 ± 0.2 µM (Fig. 5F; *n* = 6). These data indicate that VX-659 inhibits all members of the KCNN gene family with a similar affinity, whereas VX-445 exhibits selectivity for KCa3.1 over KCa2.x channels.

## Discussion

Over the past 15 years, the most significant advancement in CF therapeutics has been the development of CFTR potentiators and correctors. Indeed, Trikafta and previous generations of highly effective modulator therapies (HEMT) have resulted in diminished morbidity as well as vast improvements to quality of life of patient with CF. Surprisingly, we recently identified a novel function for C2 correctors. That is, both VX-445 and VX-121 potentiate the large-conductance Ca^2+^-activated potassium channel BK_Ca_ (KCa1.1) (14). It has been postulated that potentiating apically localized BK_Ca_ channels would be therapeutically beneficial, via an increase in the driving force for Cl^−^ secretion. Based on this, we set out to test the effects of VX-445—the current SOC C2 corrector—on forskolin-evoked Cl^−^ currents from HBEs. While the acute addition of CFTR potentiators, such as VX-770 (ivacaftor), has been well studied (8), the short-term effects of CFTR correctors on Cl^−^ secretion, in vitro, have not been explored. Here, we report the paradoxical inhibition of forskolin-mediated Cl^−^ secretion in WT and F508del CFTR-expressing HBEs in response to the acute application of the C2 CFTR corrector VX-445. In contrast, the structurally distinct C1 corrector, VX-661, which is also a component of Trikafta, does not acutely diminish Cl^−^ secretion (Fig. 1).

The surprising inhibition of Cl^−^ secretion by the acute addition of CFTR correctors begged the question: what was responsible for this paradoxical effect? We previously demonstrated that the DCEBIO-induced Cl^−^ secretory response across HBEs was due to direct activation of the basolateral membrane, Ca^2+^-activated K^+^ channel, KCa3.1 (syn. IK1) (12, 13, 35). More recently, we reported that the forskolin-stimulated Cl^−^ secretion across WT and F508del CFTR-corrected HBEs is similarly dependent upon KCa3.1 (12). Based on this, we proposed that the inhibition of KCa3.1 by VX-445 was responsible for the decreased Cl^−^ secretion observed. This hypothesis was confirmed via both whole-cell and excised patch-clamp recordings in HEK cells heterologously expressing KCa3.1. That is, VX-445, VX-659, and VX-121 inhibited KCa3.1 with low micromolar affinity (Figs. 3 and 4). In contrast, the structurally distinct C1 CFTR correctors, VX-661 and VX-809, failed to inhibit KCa3.1 at 10 µM. The most parsimonious explanation for these results is that VX-445, VX-659, and VX-121 directly associate with KCa3.1, resulting in channel inhibition.

As noted, KCa3.1 belongs to the KCNN gene family, which contains three additional members, the small conductance, Ca^2+^-activated K^+^ channels, KCa2.1–KCa2.3 (syn. SK1–SK3) (38). Importantly, KCa3.1 is readily distinguished from KCa2.x channel members based on blocker pharmacology (39). That is, KCa3.1 is blocked by charybdotoxin (40–42), clotrimazole-like compounds, including TRAM-34 (43–45), and fatty acids (e.g. arachidonic acid) (46). In contrast, the KCa2.x channels are blocked by apamin (47–49). Each of these blockers works by binding to the outer vestibule of the channel pore (e.g. charybdotoxin and apamin) or within the pore itself (e.g. TRAM-34, arachidonic acid). Indeed, the similarity of these channels outside of the pore is demonstrated by the observation that the blocker sites for TRAM-34 and arachidonic acid can be swapped between these channel family members without altering other aspects of channel function (45, 46). Based on this, we assessed the selectivity of VX-445 and VX-659 among the additional gene family members, KCa2.2 and KCa2.3. We demonstrate that VX-659 inhibits each of these channels with similar affinities (Figs. 3–5), whereas VX-445 displays a clear selectivity for KCa3.1 over KCa2.x family members (Figs. 3–5). To our knowledge, this is the first demonstration of these CFTR correctors directly influencing the gating of the KCa3.1/KCa2.x channels.

Given our data demonstrating that C2 correctors directly inhibit KCa3.1, KCa2.2, and KCa2.3, it is important to consider what this might mean in a broader context. We demonstrate that VX-445 inhibits Cl^−^ secretion across HBEs. Thus, the possibility exists that a fraction of the potential Cl^−^ secretion, which has been restored in patients with CF by Trikafta, is being blunted by the inhibition of KCa3.1. In other words, by limiting potassium efflux via inhibition of KCa3.1 from the basolateral membrane, VX-445 would decrease the driving force for Cl^−^ efflux and hence the overall Cl^−^ secretory current—impacting the efficacy of CFTR correction. In contrast, VX-445 did not inhibit the acetazolamide-dependent ${\mathrm{HCO}}_{3}^{-}$ current (Fig. 1E and F), which our data suggest is independent of KCa3.1. Importantly, clinical trials of patients taking ETI have demonstrated heterogeneity in the response to sweat chloride and ppFEV1, although the reasons for this are poorly understood (24, 50, 51).

While the current HEMT have proven to be highly efficacious in improving lung function as well as quality of life in the majority of patients with CF (52), it is important to point out that in a subset of patients AE have been reported. Critically, mental status changes have been reported in response to Trikafta (26, 31, 53–55), which has been observed in patients with no otherwise known mental disorders (27, 29). Indeed, there is some indication that HEMT worsen depression and anxiety symptoms (30, 56). However, a meta-analysis failed to find a causal link between ETI therapy and depression-related symptoms (57). Nevertheless, clinicians still consider the neuropsychiatric adverse effects of CFTR modulators as deserving a serious research effort (58), which has led some patients to discontinue treatment (29). Adjustments to dosage have been put forth as a compensatory measure to deal with mental health side effects in CF (31), and clinicians have stressed the importance of tailoring dosages to the needs of individuals’ mental health (59). Furthermore, as attempted suicide has been reported in patients on Trikafta, clinicians have recommended close monitoring following initiation of Trikafta (25). Very recently, we demonstrated that VX-445 and VX-121 directly potentiate BK_Ca_ channels, which play a critical role in the neuronal fast afterhyperpolarization, resulting in a significant effect on action potential firing frequency in primary hippocampal and cortical neurons. Based on this, we proposed that this may be associated with the AE reported. Importantly, KCa2.X channels similarly play a crucial role in the brain, where they are responsible for the medium afterhyperpolarization of the action potential (47 ). Generally speaking, activation of KCa2.x channels dampens neuronal excitability, whereas inhibition increases neuronal excitability (60). However, our data suggest that VX-445 has a poor affinity for KCa2.x channels, and this may not play a role in the neuronal effects observed. However, considering the long-established role of KCa2.x channels in the CNS, it is important to carefully consider avoiding off-target effects on these channels.

Both symptomatic hypertension and asymptomatic hypertension have also been a feature of clinical trials involving CF correctors (27, 28). Importantly, KCa3.1 is expressed in vascular endothelia, where it plays a pivotal role in mediating the endothelial-derived hyperpolarization response—a nitric oxide–independent mechanism for vasorelaxation. For example, activation of KCa3.1 results in decreased blood pressure (BP) (61), while knockout of KCa3.1 results in an increase in BP (62). KCa2.3 is also expressed in vascular endothelia, and just as is the case for KCa3.1, knockdown of KCa2.3 increases BP in mice, whereas overexpression decreases BP (61, 62). Consistent with this, pharmacological activation of KCa2.3, together with KCa3.1, results in vasorelaxation and a decrease in BP (61). Given the role of KCa3.1 and KCa2.3 in modulating vascular tone and hence BP, it is possible that hypertensive events occurring in patients with CF are the result of KCa3.1 and KCa2.X inhibition in vascular endothelia. However, inhibition of KCa3.1 and KCa2.X could be overridden by the activation of BK_Ca_ (KCa1.1), and we have previously shown that VX-445 is capable of relaxing preconstricted mesenteric artery (14).

In summary, we report a paradoxical C2 CFTR corrector-dependent decrease in Cl^−^ secretion in WT CFTR HBEs, which we attribute to the direct inhibition of KCa3.1. We further show that CFTR correctors demonstrate affinities for KCa channels in the low micromolar range. Thus, it is important to consider how these concentrations relate to known concentrations of CFTR correctors in patients with CF. In this regard, clinical trial data demonstrate maximal (*C*_max_) and minimal (*C*_min_) plasma concentrations of VX-445 between 8.4 and 9.2 µg/mL (∼15 µM) and 4.0 and 5.4 µg/mL (∼6–9 µM), respectively (20). Thus, the apparent *K*_i_s we determine for these KCa channels are clearly observed in plasma, suggesting that these KCa channels may be targeted by CFTR correctors, particularly in the vasculature. However, it is important to recognize that drugs are transported in the blood bound to albumin with affinities that allow them to rapidly dissociate and bind to their intended target (63). For example, binding with a 1-µM affinity would indicate an off-rate of <1 sec. In this regard, we demonstrate that the inhibition of KCa3.1 by VX-445 was independent of albumin (3.4%), indicating that VX-445 can freely dissociate and bind to KCa3.1. Whether the effects we observe on KCa3.1 in HBEs, or KCa3.1/KCa2.x channels in heterologous expression systems, translates to effects on other cell/tissue types (e.g. ex vivo intact vasculature or neurons) to alter their physiological function is the focus of current investigations. A further question, which has only recently begun to receive attention, is what are the concentrations of CFTR correctors and potentiators that are achieved in cells in vivo? In this regard, the CFTR potentiator VX-770 was shown to accumulate in vivo in nasal epithelia from patients, as assessed following brush biopsy (64). In a follow-up study, these authors compared plasma concentrations with nasal epithelial biopsies and find that they do not correlate and are highly variable, ultimately concluding that some patients maintain a concentration of drug largely in excess of that necessary for clinical benefit (65). More recently, these studies were extended to elexacaftor (VX-445) where it was found that the mean concentration of VX-445 in nasal brushing cell lysates from patients with CF ranged from 0 to 5454 ng/mL (∼9 µM) (23). Thus, the concentration of VX-445 achieved in cells is within the range we determined for the *K*_i_ of KCa3.1/KCa2.x channels. In the short term, one strategy for mitigating AE caused by inhibition of KCa channels may be to decrease the dose of CFTR correctors, and this strategy has been employed in attempts to ameliorate mental status effects (29, 31). While the clinical benefit of current HEMT is beyond dispute, in the long-term, our results may help in guiding the development of the next generation of CFTR correctors that correct CFTR folding/trafficking while not targeting KCa channels, thus further improving the lives of patients with CF.

## Materials and methods

### Sex as a biological variable

Our study did not consider sex as a biological a variable as the authors were blind to the sex of human donor tissue.

### Cell culture

HEK cells stably expressing KCa3.1 (IK1, SK4), KCa2.3 (SK3), and KCa2.2 (SK2) were generated and cultured as previously described by us (37, 66–71).

Primary human bronchial epithelial cells (HBEs) were collected from unused or discarded samples from the Center for Organ Recovery and Education via the University of Pittsburgh Pulmonary, Allergy, Critical Care, and Sleep Medicine Lung Biobank and Repository. All experimental procedures involving the use of HBEs were approved by the institutional review board (IRB STUDY19090084) and the Committee for Oversight of Research and Clinical Training Involving Decedents (CORID number 451) at the University of Pittsburgh. At all times, authors were blind to the identity of HBE donors. HBEs were cultured using the Vertex method (72). Studies were carried out on WT CFTR HBEs from 11 (13) donors and homozygous F508del/F508del CFTR HBEs from five (5) separate donors. HBEs were plated on Costar Transwell permeable supports (0.4 μM pore size, 6.5 mm insert, polyester membrane, Corning) and grown at an air–liquid interface for 5+ weeks in HBE differentiation medium containing 2% Ultroser-G. Basolateral media were replaced three times per week. For whole-cell patch-clamp studies, HBEs were maintained in BronchiaLife airway media (LifeLine Cell Technology, LS-1047) and plated onto poly-L-lysine-coated coverslips (see below).

### Ussing chamber *I*_sc_ measurements

Costar Transwell inserts were mounted in a modified Ussing chamber (P2300, Physiologic Instruments) and the monolayers continuously short-circuited (VCC MC8, Physiologic Instruments) by forcing the transepithelial voltage to 0 mV. Short-circuit current (*I*_sc_) measurements were taken in symmetric solutions, containing (in mM): 120 NaCl, 25 NaHCO_3_, 3.3 KH_2_PO_4_, 0.8 K_2_HPO_4_, 1.2 CaCl_2_, 1.2 MgCl_2_, and 10 glucose. The pH of the solution is 7.4 when gassed with 95% O_2_–5% CO_2_ at 37°C. Compounds were added cumulatively following the establishment of a new stable current response. Amiloride was added to the apical membrane, while all other compounds were added to both membranes due to their lipophilic nature. In all experiments, 10 µM amiloride was used to inhibit sodium absorption. Percent inhibition was calculated as the change in *I*_sc_ (Δ*I*_sc_) between the forskolin or DCEBIO stable current level and the *I*_sc_ following addition of the compound being assessed divided by the Δ*I*_sc_ between the stable forskolin- or DCEBIO-induced *I*_sc_ and the *I*_sc_ following bumetanide addition.

### Patch-clamp electrophysiology

HEK cells expressing the channel of interest were plated onto poly-L-lysine-coated glass coverslips 1–2 days prior to patch-clamp analysis. Electrophysiological recordings in the excised, inside-out, and whole-cell patch-clamp configurations were performed as recently described (14). For excised patch-clamp recordings, the pipette solution contained (in mM): 140 K-gluconate, 5 KCl, 1 CaCl_2_, 1 MgCl_2_, and 10 HEPES. The pH was adjusted to 7.2 with KOH. The bath solution contained (in mM): 145 K-gluconate, 5 KCl, 1.3 MgCl_2_, 10 HEPES, and 1 EGTA. Sufficient CaCl_2_ was added to obtain the desired free Ca^2+^ concentration at a pH of 7.2 (adjusted with KOH). When recording KCa3.1 channels, ATP (300 µM) was added to the bath to diminish channel rundown (73). All recordings were carried out at a voltage of −100 mV, referenced to the extracellular compartment, as is standard. The apparent *K*_i_ and Hill coefficient for each CFTR corrector on KCa3.1, KCa2.3, and KCa2.2 were determined during a continuous recording by step-wise addition of increasing concentrations of compound and fitting the resultant relative remaining current to a Hill equation (Prism v. 10.1.0). The zero current level was determined by the addition of a 0 Ca^2+^ solution (zero-added Ca^2+^ plus 1 mM EGTA) at the end of the experiment. In the case of KCa3.1, TRAM-34, a known inhibitor of KCa3.1 (45), was used to confirm expression of the channel. For whole-cell experiments, pipettes contained (in mM): 145 K-gluconate, 10 EGTA, 7.5 CaCl_2_, 2 MgCl_2_, 3 mM NaATP, and 10 HEPES. The bath solution contained (in mM): 140 K-gluconate, 5 KCl, 1.0 MgCl_2_, 10 HEPES, and 2 CaCl_2_. Current–voltage relationships were determined by stepping from a holding potential of −80 mV to +80 mV in 20 mV increments for 400 ms. The middle 300 ms of each trace was selected to avoid any influence of capacitive transients, and average currents were calculated for each voltage via the *I–V* tool in *Clampfit*. To control for cell size, whole-cell currents were normalized to cell capacitance.

### Chemicals

Amiloride, TRAM-34, acetazolamide, and bumetanide were obtained from Sigma-Aldrich. Forskolin was obtained from LC Laboratories. 5,6-Dichloro-1-ethyl-1,3-dihydro-2H-benzimidazol-2-one (DCEBIO) was synthesized in the Bridges Lab, as described (10). VX-445, VX-121, VX-659, VX-661, VX-809, and VX-770 were obtained from MedChemExpress. Ultroser-G was obtained from Pall Life Sciences (Cergy-Saint-Christophe, France).

### Statistics

All data were presented as means ± SEM, where *n* indicates the number of filters or patch-clamp recordings. We assessed whether the data were normally distributed using both the D’Agostino and Pearson omnibus normality test and the Shapiro–Wilk normality test in GraphPad Prism (v. 10.1.0). Comparisons between two experimental maneuvers within an experiment were assessed for significance using a paired Student's t test, as were comparisons of varying concentrations of drug with control, when the number of experimental maneuvers differed among concentrations tested. Significance between experiments was determined by an unpaired t test. Significance between multiple experimental maneuvers within an experiment was determined by an ANOVA followed by a Tukey's honestly significant different post hoc test. All statistical analysis was carried out using GraphPad Prism (v. 10.1.0). A value of *P* < 0.05 was considered statistically significant and reported.

## Supplementary Material

pgaf211_Supplementary_Data

## Data Availability

Raw data values are provided in the supplementary data value file, and all data are included in the manuscript and/or supporting information.
